# Role of Raf-like kinases in SnRK2 activation and osmotic stress response in plants

**DOI:** 10.1038/s41467-020-19977-2

**Published:** 2020-12-03

**Authors:** Norma Fàbregas, Takuya Yoshida, Alisdair R. Fernie

**Affiliations:** Max Planck Institute of Molecular Plant Physiology, Am Mühlenberg 1, 14476 Potsdam-Golm, Germany

**Keywords:** Plant signalling, Drought

## Abstract

Environmental drought and high salinity impose osmotic stress, which inhibits plant growth and yield. Thus, understanding how plants respond to osmotic stress is critical to improve crop productivity. Plants have multiple signalling pathways in response to osmotic stress in which the phytohormone abscisic acid (ABA) plays important roles. However, since little is known concerning key early components, the global osmotic stress-signalling network remains to be elucidated. Here, we review recent advances in the identification of osmotic-stress activated Raf-like protein kinases as regulators of ABA-dependent and -independent signalling pathways and discuss the plant stress-responsive kinase network from an evolutionary perspective.

## Abstract (web summary)

A better understanding of how plants respond to osmotic stress could potentially help improve crop yields. Here Fàbregas et al. review the recent characterization of Raf-like kinases that act in both in ABA-dependent and -independent responses to osmotic stress.

## Introduction

Environmental water-deficit stresses such as drought, cold, and salinity impose osmotic stress on plants, leading to reduced plant growth and loss of yield. To protect themselves from drought, plants need to sense osmotic stress and trigger the signal transduction pathways that activate the adaptation of physiological mechanisms used by plants to adapt to water-deficit stress. Thus, understanding how plants respond to water-deficit stress is critical both to improve plant stress tolerance and ultimately to increase crop productivity in the field. When plant cells sense osmotic stress caused by drought and high-salinity stress, the levels of the key phytohormone, abscisic acid (ABA), increase dramatically and, subsequently, the ABA signalling pathway is activated in the plant^1–3^. Despite being a key signalling molecule, ABA is not highly accumulated under several osmotic stress conditions, such as mild-salinity^4,5^ and low temperature^6,7^. The term osmotic stress will hereafter indicate the stress caused by drought or high-salinity stress. To date, the molecular mechanisms used by plants for early events of osmotic stress signalling, including osmosensing, have not been completely revealed.

The molecular mechanisms of plant responses to osmotic stress were initially revealed via the genetic studies of the model plant *Arabidopsis thaliana* (*Arabidopsis*) that identified the mutants showing altered sensitivity to salt stress^8,9^ and ABA^10–12^. The characterization of *cis*-elements in the promoters of stress-inducible genes (reviewed in refs. ^13,14^) also led to the identification of key transcription factors. Consistent with reports showing that the ABA-responsive elements (ABREs) are enriched in the dehydration- or drought-inducible genes in *Arabidopsis*, rice, soybean, and poplar^15,16^, the bZIP transcription factors targeting ABREs, called ABRE-BINDING proteins/ABRE-BINDING FACTORs (AREB/ABFs), play key roles in ABA-dependent gene expression under osmotic stress conditions in several species^17^. The dehydration-responsive element (DRE)/C-repeat, another key *cis*-element in response to drought, heat, and cold stresses, is recognized by the AP2/ERF transcription factors, DRE-BINDING proteins (DREBs)^18^ or C-REPEAT/DRE-BINDING FACTORs^13,14^. In particular, DREB2A, the expression of which is not induced by ABA, plays key roles in osmotic- and heat-stress responses in *Arabidopsis*^19,20^. On the basis of the extensive studies on these transcription factors, AREB/ABF and DREB2A signalling pathways are thought to be pivotal in plant responses to osmotic stress^21^.

However, as demonstrated in the transcriptional change in response to ABA^22^, the gene expression under osmotic stress conditions is dynamically regulated by a myriad of *cis*-elements and transcription factors, and not these pathways alone. Epigenetic regulations, including DNA methylation, histone modifications, and nucleosome assembly by chaperones, also play important roles in osmotic-stress responses in plants and are well summarized in other reviews^23–25^.

Osmotic stress generated by drought and high-salinity conditions promotes the accumulation of ABA in plant cells and the activation of the ABA signalling pathway. The core molecular components of ABA perception and signal transduction have been well studied^1–3^. Following prior studies of sucrose nonfermenting-1-related protein kinases 2 (SnRK2s) in several species, including *Arabidopsis*^26–28^, *Vicia faba*^29^, and barley^30^, three *Arabidopsis* SnRK2s activated by ABA were shown to be important components of the ABA signalling pathway^31–33^. Under control conditions, when plants are growing in well-watered environments, the ABA-dependent SnRK2s are inhibited by clade A protein phosphatases of type 2C (PP2C)^34,35^, keeping the ABA signalling pathway inactive. Under specific water-deficit stress conditions, ABA levels increase and it binds to the receptor proteins, designated as PYRABACTIN RESISTANCE 1 (PYR1), PYR1-LIKE (PYL), or REGULATORY COMPONENTS OF ABA RECEPTOR (RCAR)^36,37^. The receptor complexes of PYR/PYL/RCARs and PP2Cs, which serve as coreceptors, are formed, allowing the activation of the SnRK2s proteins. Once released from PP2C, SnRK2s auto-phosphorylate and self-activate^38,39^, subsequently activating or inhibiting a group of downstream transcription factors, including AREB/ABFs, and membrane proteins, including SLOW ANION CHANNEL-ASSOCIATED (SLAC1), via phosphorylation. Given that a large majority of the downstream genes of the SnRK2s were downregulated in the multiple *areb/abf*-knockout mutant^40^, AREB/ABFs have been suggested to be the key transcription factors in the ABA core signalling. Consistently, AREB/ABFs are among the top contributors in the hierarchical gene regulatory network in response to ABA^22^.

Although ABA is the key signalling molecule in response to osmotic stress, osmotic stress additionally activates signalling pathways even if ABA signalling is off. For example, the DRE/C-repeat *cis*-element has been shown to be crucial for the gene induction independently of ABA, as evidenced by the dehydration-responsive expression of *RESPONSIVE TO DESICCATION 29A* (*RD29A*) in the *aba insensitive 1* (*abi1*) mutant^41,42^, the genetically isolated dominant mutant of clade A PP2C. Taking advantage of the genetic resources, in which ABA metabolism, signalling, or transport are impaired, the signalling pathway has been well studied. In contrast to the well-described ABA signalling pathway, an ABA-independent signalling pathway is activated in response to osmotic stress in plants as well, yet the molecular components of this osmotic stress-signalling pathway and the early osmosensing mechanisms used by plants are still not well understood. A plasma membrane protein that forms a calcium-permeable channel sensitive to osmotic stress named OSCA1 (REDUCED HYPEROSMOLARITY INDUCED Ca^2+^ INCREASE 1) was proposed as a plausible sensor of rapid calcium signalling activated by osmotic stress^43^. However, whether OSCA1 is the responsible protein for directly sensing the osmotic stress in the plant cells has not been fully demonstrated. Given that ARABIDOPSIS HISTIDINE KINASE 1 (AHK1) complemented the high-salinity-sensitive phenotype of the *Saccharomyces cerevisiae* (yeast) mutant lacking osmosensors, AHK1 was also proposed to sense and transduce osmotic-stress signals^44,45^. By contrast, AHK1 was reported to be not involved in a rapid stomatal closure or ABA biosynthesis in response to decrease of air humidity^46^ and thus further experimental validations in plants are required. Similarly, the upstream molecular components of the signalling pathways regulating the DREB2A transcription factor in response to osmotic stress remain unknown. Even in the absence of osmotic stress, *DREB2A* is expressed and the DREB2A protein is degraded via ubiquitin–proteasome pathways, preventing activation of the stress signal^47,48^, implying that stress-responsive stabilization and activation mechanisms exist. In heat-stress responses, DNA POLYMERASE II SUBUNIT B3-1 (DPB3-1)/NUCLEAR FACTOR Y, SUBUNIT C10 (NF-YC10) was isolated as an interacting protein of DREB2A and the NF-Y protein complex has been suggested to regulate the target selectivity of DREB2A. However, DPB3-1/NF-YC10 was found to be unlikely involved in drought or high-salinity stresses^49^. Despite this insight, our current knowledge on the molecular mechanisms of osmotic stress signalling pathways in plants is still limited. In particular, little is known about key components in the early events and, therefore, the global osmotic stress signalling network remains to be elucidated.

Genetic approaches have been playing a fundamental role in exploring key genes of osmotic stress and ABA signalling pathways. Indeed, a chemical genetic screening has identified the ABA receptor proteins^36^. Alongside developments of genomics and transcriptomics in the early 2000s, the focus of reverse-genetic studies was mainly on transcriptional changes in response to stresses. However, the cellular responses to stress occur more quickly through post-transcriptional and post-translational modifications, including phosphorylation. Nowadays, high-throughput proteomic analyses based on mass spectrometry (MS) are available, enabling us to investigate phosphoproteomic changes in response to short-term osmotic stress and to explore the interaction of proteins with known key regulators. Here, we review the most recent advances in the identification of new molecular components with essential roles in the ABA-dependent and -independent signalling pathways in response to osmotic stress. Five independent studies have recently described different families of Raf-like protein kinases, which are activated early in response to osmotic stress and operate upstream of both the ABA-dependent and -independent signalling pathways in *Physcomitrella patens* (*P. patens*)^50^ and *Arabidopsis*^51–54^. We aim to connect these novel findings concerning the different families of Raf-like protein kinases with state-of-the-art knowledge in the field to obtain a general schematic picture of the osmotic regulation of ABA-dependent and -independent pathways. We further discuss the stress-responsive kinase network in plants from an evolutionary perspective.

### SnRK2s contributes to ABA-dependent and -independent signalling pathways

The SnRK2 family of proteins contains ten members in *Arabidopsis*, which can be divided into three subclasses I, II, and III on the basis of their sequence homology and responsiveness to osmotic stress and ABA^17,55^. For the clarity of the readers, the different nomenclatures used by the different studies reviewed herein are summarized and classified in Supplementary Table 1. In this review, we mainly use the alphabetical nomenclature rather than the numerical one (e.g., SRK2G instead of SnRK2.1).

**Fig. 1:**
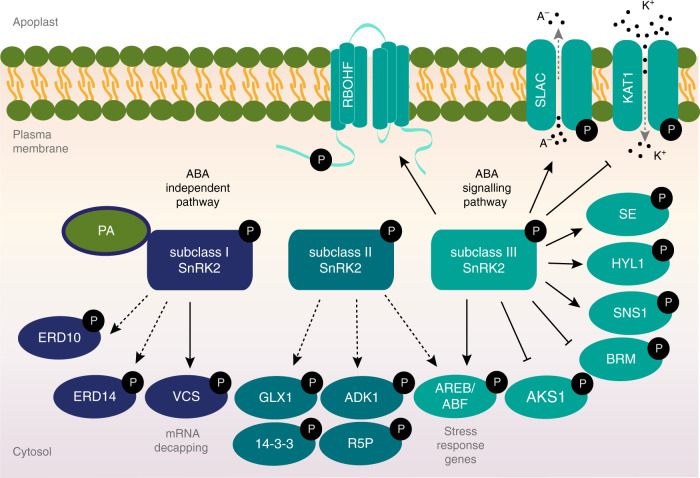
SnRK2s downstream targets in osmotic stress signalling pathways. Schematic cartoon of the downstream targets described for the different subclasses of SnRK2s. ABA-unresponsive subclass I SnRK2s are activated by phosphorylation under osmotic stress and activate components of the mRNA decapping complex VCS through direct phosphorylation. Subclass I SnRK2s also interact with PA and phosphorylate two dehydrin proteins, ERD10 and ERD14, in response to osmotic stress. ABA-responsive subclass II SnRK2s are activated by phosphorylation under osmotic stress and phosphorylate GLX1, 14-3-3, ADK1 and R5P target proteins. Subclass II SnRK2s likely activate AREB/ABF transcription factors in response to osmotic stress. Subclass III SnRK2s, the core components of ABA signalling, are activated by phosphorylation under osmotic stress and they phosphorylate AREB/ABF, which in turn activate the expression of stress-responsive genes. Subclass III SnRK2s also phosphorylate a number of substrates, including AKS1 transcription factors, BRM, SNS1, HYL1, and SE1 proteins and SLAC1, RBOHF, and KAT1 membrane proteins. PA, phosphatidic acid; VCS, VARICOSE; ERD, EARLY RESPONSIVE TO DEHYDRATION; GLX1, GLYOXALASE 1; ADK1, ADENOSINE KINASE 1; R5P, Ribose 5-phosphate isomerase; AKS1, ABA-RESPONSIVE KINASE SUBSTRATE 1; BRM, BRAHMA; SNS1, SNRK2-SUBSTRATE 1; HYL1, HYPONASTIC LEAVES; SE, SERRATE; MAPKKK, mitogen-activated protein kinase kinase kinases; SLAC, SLOW ANION CHANNEL-ASSOCIATED; A^−^, anions; KAT1, POTASSIUM CHANNEL IN ARABIDOPSIS THALIANA 1; K^+^, potassium; RBOHF, RESPIRATORY BURST OXIDASE HOMOLOG F. Straight arrows indicate direct interaction and phosphorylation, dashed arrows (black) indicate in vitro interactions not fully experimentally validated in vivo, and dashed arrows (grey) depict ion flux through membrane channel proteins.

Given that they are activated by ABA, subclass II and III are classified as ABA-dependent SnRK2s (Supplementary Table 1). In *Arabidopsis*, SRK2E (also known as OST1) was first functionally characterized as a key regulator of ABA signalling in guard cells by genetic screening^26^ and biochemical analyses^28^. Consistent with the strong activation by ABA^55^, the *knockout* mutants of three subclass III SnRK2 members, SRK2D, SRK2E, and SRK2I, showed extreme insensitivity to ABA, and it has been revealed that they play an essential role in the ABA signal transduction pathway^31–33^. Among varieties of their substrates, AREB/ABFs are key transcription factors phosphorylated and activated by the SnRK2s in response to ABA^33,56^ (Fig. 1) with the SnRK2-AREB/ABF signalling pathway also being reported in rice^57^. By contrast, ABA-RESPONSIVE KINASE SUBSTRATE 1, also known as FLOWERING bHLH 3, is phosphorylated and inactivated by SRK2E in guard cells^58^ (Fig. 1). SRK2E additionally phosphorylates membrane proteins essential for ABA-mediated stomatal closure response following water-deficit stress, namely SLAC1 and POTASSIUM CHANNEL IN ARABIDOPSIS THALIANA 1 (KAT1)^59–61^ (Fig. 1). SLAC1 channel activity was shown to be tightly controlled via phosphorylation and dephosphorylation. In the absence of ABA, SLAC1 is inhibited by phosphatases, including ABI1 and ABI2^62^, whereas SLAC1 is activated in the presence of ABA via phosphorylation by SRK2E and the calcium-dependent protein kinases (CPKs), such as CPK6, CPK21, and CPK23^59,60,62^. Moreover, the NADPH oxidase, RESPIRATORY BURST OXIDASE HOMOLOG F (RBOHF), which is responsible for ABA-induced ROS production and subsequent stomatal closure, as well as RBOHD^63^, has been shown to be phosphorylated by SRK2E^64^. Collectively, SRK2E promotes stomatal closure through activation of SLAC1, inhibition of KAT1, and activation of RBOHF to induce ROS production (Fig. 1). Furthermore, phosphoproteomic analyses identified putative targets of subclass III SnRK2s proteins, which are involved in transcription, RNA processing, epigenetic modifications, chloroplast processes, and control of flowering^65,66^. SNRK2-SUBSTRATE 1 was suggested to function as a negative regulator of ABA signalling^66^, whereas SERRATE and HYPONASTIC LEAVES, which are involved in microRNA biogenesis, were shown to be phosphorylated by the SnRK2s^67^. The SWI2/SNF2 chromatin remodelling ATPase BRAHMA was also reported to be inhibited via phosphorylation by the SnRK2s in response to ABA (Fig. 1) and activated via dephosphorylation by the clade A PP2Cs^68^. For an extensive review on previously described substrates and functions of the subclass III SnRK2s and their functional relationship with calcium signalling, please see refs. ^56,69,70^.

Subclass II members, SRK2C and SRK2F, are activated by osmotic stress (Supplementary Table 1) and show a weaker activation by ABA than the subclass III SnRK2s^55^. This result appears to be consistent with the fact that ABA-responsive SnRK2s of subclasses II and III harbour the acidic Asp-rich domain at the C terminus^17^, which nearly corresponds to the ABA box essential for the interaction with PP2C^38^. However, as the triple mutant of subclass III SnRK2s shows extreme ABA insensitivity^33^, the role of subclass II SnRK2s in ABA signalling is thought to be minor. Notably, rice subclass III SnRK2s, but not subclass II SnRK2s, were shown to be activated by ABA^71^. SRK2C mainly expressed in root tips has been shown to be involved in enhancement of drought tolerance^72^ and vegetative growth^73^ in *Arabidopsis*. By contrast to the phenotypic changes exhibited by the overexpressing lines^72,73^, the single and double mutants of *SRK2C* and *SRK2F* did not show altered growth phenotypes^74^. The downstream substrates of SRK2C has been suggested to include AREB/ABF transcription factors, such as ABF3^74^ (Fig. 1). Furthermore, a phosphoproteomic approach identified candidate target proteins of SRK2C associated with metabolic processes, such as 14-3-3 proteins, glyoxalase 1, adenosine kinase 1, and ribose 5-phosphate isomerase^73^ (Fig. 1). However, further investigation of the candidate substrates of subclass II SnRK2s will be required to reveal their biological functions under osmotic stress conditions.

Given that SnRK2s from subclass I, with the exception of SRK2J, are activated by osmotic stress but not ABA^55^, SRK2A, SRK2B, SRK2G, and SRK2H (Supplementary Table 1) are potentially involved in the ABA-independent osmotic-stress signalling pathway. Compared to subclass III SnRK2s, until recently little was known about the activation mechanisms, targets and interactors of subclass I SnRK2s^75–77^. SRK2A and SRK2B are involved in maintenance of root growth and development^76^ and ROS homoeostasis^78^ in response to salt stress. In addition to their activation within 30 s of salt treatment^76^, SRK2A localized at the primary root tip displayed a punctuate pattern under salt stress conditions. However, a similar pattern was not observed for SRK2B localized in the vascular tissues at the site of the lateral roots^76^. Furthermore, fractionation experiments of SRK2A and SRK2B indicated that these cytosolic kinases are associated to the membrane in salt stress conditions and interact with phosphatidic acid^76,79^ (Fig. 1). By contrast to ABA-dependent SnRK2s, no transcription factors have been characterized to be substrates of subclass I SnRK2s. However, co-immunoprecipitation analyses detected different components of the mRNA decapping complex, such as VARICOSE (VCS) and DECAPPING 2 (DCP2), as candidate interactors of SRK2G^77^. The mRNA decapping protein complex is responsible for removal of the 5′-cap, a critical step for exonuclease-mediated mRNA decay^80^. The core decapping complex is formed by DCP2, the active enzyme of the decapping complex, DCP1, and the scaffold protein VCS, and it assembles in processing bodies (P-bodies) in the cytoplasm^80^. Bimolecular fluorescence complementation and phosphorylation assays confirmed that the subclass I SnRK2s directly interact and phosphorylate VCS^77^ (Fig. 1). As well as SRK2A^76^, SRK2G also showed the translocation from cytosol to P-body in response to osmotic stress but not ABA, corroborating the interactions of VCS with SRK2A and SRK2G in P-bodies^77^. Moreover, mRNA decay was shown to be impaired in the *srk2a srk2b srk2g srk2h* quadruple mutant and the VCS artificial microRNA (amiRNA) line, both of which showed growth retardation under osmotic stress conditions, indicating that subclass I SnRK2s are involved in mRNA metabolism via phosphorylation of VCS, and that this process appears to be important for shifting mRNA population in response to osmotic stress. As no interactions were detected between VCS and ABA-responsive SnRK2s^77^, ABA-unresponsive SnRK2s might exclusively regulate VCS, yet it has been recently reported that SRK2E was able to phosphorylate VCS in vitro^81^. Another recent phosphoproteomic study suggested that SRK2B phosphorylates two LATE EMBRYOGENESIS ABUNDANT/dehydrin proteins, EARLY RESPONSIVE TO DEHYDRATION 10 and 14, in response to osmotic stress^82^ (Fig. 1). Further in vivo studies need to be carried out to identify the molecular components regulated by the subclass I of SnRK2s. In conclusion, different studies have supported that osmotic stress-activated subclass I SnRK2s play different roles in plants than ABA-responsive SnRK2s.

### Novel roles for Raf-like kinases in osmotic stress pathways upstream of the SnRK2s

With the exception of a few examples, such as *Arabidopsis* SRK2J, SnRK2 protein kinases were reported to be activated by osmotic stress in *Arabidopsis*^55^, rice^71^, soybean^83^, tobacco^84^, and *P. patens*^85^. Although a subset of them grouped into subclass II and III were also activated in response to ABA^55,71,85^, the activation by osmotic stress still occurred in ABA signalling mutants where the ABA-induced activation is abolished^86–88^, implying that both ABA-dependent and -independent signal transduction cascades are involved. SnRK2s are suggested to be auto phosphorylated^38,39^, although structural analysis^39^ revealed that the activation loop of SRK2E is more stable than that of SRK2D and SRK2I, implying that the level of autophosphorylation might differ among the SnRK2s. By contrast, previous pharmacological studies demonstrated that the general kinase inhibitor staurosporine inhibits the SnRK2s’ kinase activities in vitro but not their activation by osmotic stress or ABA treatment^84,86^, suggesting that the osmotic-stress responsive SnRK2 activation requires upstream protein kinases that are insensitive to staurosporine. A pioneering study in *P. patens*^50^ and more recent four independent ones in *Arabidopsis*^51–54^ have identified a family of protein kinases that are activated in response to osmotic stress and subsequently phosphorylate and activate SnRK2s.

**Fig. 2:**
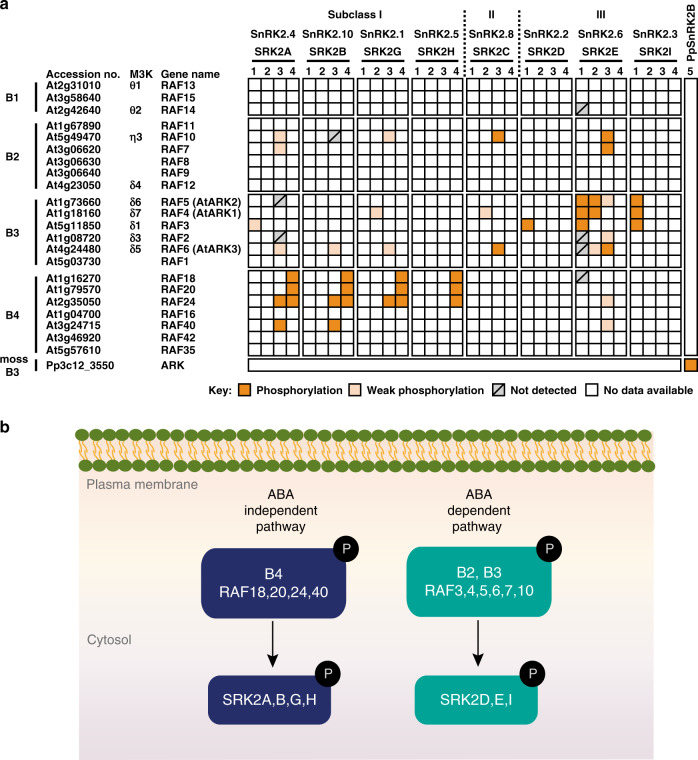
Subgroup B Raf-like MAPKKKs and their substrate specificities for SnRK2 kinases. **a** Heat map shows the in vitro phosphorylation of SnRK2 kinases by Raf-like MAPKKKs of *Arabidopsis* and *P. patens*. *Arabidopsis* SRK2J (subclass I) and SRK2F (subclass II) were excluded due to no data available. According to the phylogenetic tree^33^, PpSnRK2B belongs to subclass III SnRK2s. **1**, Takahashi et al.^51^; **2**, Katsuta et al.^54^; **3**, Lin et al.^52^; **4**, Soma et al.^53^; **5**, Saruhashi et al.^50^. Please also refer to Supplementary Tables for the accession numbers and the gene names. The accession numbers of *P. patens* genes: ARK, Pp3c12_3550 (in the *P. patens* genome v.3.3), also referred to as Pp1s462_10V6 or Phypa_30352; PpSnRK2B, Pp3c6_16600, also referred to as Pp1s240_91V6 or Phypa_195464. ARK, ABA, and abiotic stress-responsive Raf-like kinase; M3K, MAP kinase kinase kinases. **b** Raf-like MAPKKKs are activated by phosphorylation under osmotic stress upstream of SnRK2s in *Arabidopsis*. B2/B3 family of Raf-like MAPKKKs regulate the ABA signalling pathway by upstream phosphorylation of the ABA-responsive subclass III SnRK2s and B4 Raf-like MAPKKKs regulate the ABA-independent signalling pathway by phosphorylation of the ABA-unresponsive subclass I SnRK2s. Schematic cartoon displays proposed signalling pathways based on in vitro assays (**a**) and detailed examinations, including in vivo phosphorylation, using the mutants of Raf-like MAPKKKs. Straight arrows indicate direct interaction and phosphorylation.

A mutagenesis screening for ABA-insensitive *P. patens* mutants revealed that a mutation in a Raf-like protein kinase cause reduced ABA sensitivity and osmotic stress tolerance^50^. The authors named this Raf-like protein ARK (ABA and abiotic stress-responsive Raf-like kinase). ARK is a single gene from the B3 Raf-like kinases family group of mitogen-activated protein kinase kinase kinases (B3-MAPKKKs) in *P. patens* and *ark* loss-of-function mutants showed decreased ABA sensitivity and cold acclimation capacity, yet increased sensitivity to osmotic stress. Moreover, ABA-responsive genes were differentially expressed in the *ark* mutant. Given that the *P. patens* SnRK2-like protein was shown to be activated by ABA in a prior study^89^, the kinase activity of the corresponding protein was examined in the *ark* mutant, in which ABA, cold, desiccation and osmotic stress treatments did not fully activate the kinase. The *ark* mutants identified in the study were not defective in the amount of ARK protein per se but rather by an impaired activity of the ARK protein due to a single C to T mutation (Ser532Phe) that impairs the ARK protein phosphorylation and causes ABA insensitivity^50^. Furthermore, ARK was shown to directly phosphorylate and activate PpSnRK2B (Fig. 2a). Specifically, ARK phosphorylated the PpSnRK2B protein on the Ser165 and Ser169 residues, the latter is corresponding to the critical residue for the activation of SnRK2s in *Arabidopsis*^86^. To be active, ARK itself needs to be phosphorylated on the Ser1029 residue^50^. However, no upstream kinase phosphorylating ARK has been identified to date.

Recently, four independent studies have also identified different groups of Raf-like protein kinases activated by osmotic stress^51–54^. All four studies converge in a common finding: the Raf-like MAPKKKs are activated in response to osmotic stress and function upstream of the SnRK2 kinases in *Arabidopsis*, as previously reported in *P. patens*^50^. The B2/B3 family of Raf-like MAPKKKs regulate the ABA signalling pathway by phosphorylation of the ABA-responsive SnRK2s from subclass III, consistent with the findings in *P. patens* (Fig. 2). By contrast, B4 Raf-like MAPKKKs regulate the ABA-independent signalling pathway by phosphorylation of the ABA-unresponsive SnRK2s from subclass I (Fig. 2). It is noteworthy that the studies discussed below use different nomenclatures, which can be confusing for the reader (Raf-like MAPKKKs, M3PK∂ kinases, OK^100^/OK^130^ kinases, or AtARKs). Therefore, for the clarity of this review, we decided to use the same nomenclature *(*Raf-like MAPKKKs) for all the studies. In addition, we have summarized and classified the different nomenclatures used within the studies reviewed herein in Supplementary Table 2.

MAPK signalling cascades play important roles in plant growth, development and stress responses. The MAPK cascade is typically composed of three kinases: MAPKKK, MAPKK, and MAPK. The MAPK cascades transfer signals through sequential phosphorylation activation steps^90^. The *Arabidopsis* genome contains 80 MAPKKKs, 10 MAPKKs, and 20 MAPKs. MAPKKKs are divided into two major groups: MEKK‐like and Raf‐like kinases^90,91^. There are 48 Raf‐like kinase grouped into three families, A, B, and C, in the *Arabidopsis* genome, yet the function of most members has not been uncovered. The family B contains 22 members and is divided in 4 subfamilies: B1 subfamily includes 3 members, B2 subfamily includes 6 members, B3 subfamily includes 6 members, and the B4 subfamily includes 7 members (Supplementary Table 2). Within the B3 subfamily, RAF1, also named constitutive triple response 1 (CTR1, Supplementary Table 2), is one of the best-characterized MAPKKKs in plants and constitutes the MAPK pathway in ethylene signalling^92^. RAF1 has been shown to play essential roles both in ethylene and ABA signalling pathways^93^ as well as in response to hypoxia^94^. The B3 family member RAF2, also named enhanced resistance (EDR. Supplementary Table 2), negatively regulates the biotic stress immune response^95^. In *Arabidopsis*, loss-of-function mutants of members of the B2 and B3 families of the Raf-like protein kinases such as RAF1, RAF10, and RAF11, are insensitive to ABA^96,97^ and the *raf5* mutant is hypersensitive to salt stress^98^. In addition, a member of the B4 subfamily, RAF18 is phosphorylated during the early stages of the osmotic stress response^99^. Although all these studies revealed important roles of Raf-like protein kinases in plants, their function in osmotic stress and ABA signalling pathways in higher plants remained unknown.

Takahashi et al.^51^ performed genetic screenings for ABA-insensitive seed germination and identified two independent amiRNA lines targeting five or seven genes of B1 and B3 Raf-like MAPKKKs. Among the overlapped targets, B3 Raf-like MAPKKKs, RAF3, RAF4, and RAF5 phosphorylated in vitro dephosphorylated-SRK2E, which had lost its autophosphorylation activity^51^. Notably, in the presence of the PYR1 receptor and HYPERSENSITIVE TO ABA 1 (HAB1) PP2C phosphatase, SRK2E was inhibited and was not reactivated by ABA addition, whereas RAF5 reactivated the SRK2E inhibited by HAB1 in response to ABA. Further reconstitution experiments in *Xenopus* oocytes confirmed that SRK2E is activated by upstream RAF3, RAF4, and RAF5 kinases (Fig. 2). The same conclusion has been reached from the analyses of Arabidopsis RAF4, RAF5 and RAF6^54^ (Fig. 2), which were able to restore the ABA insensitivity of the *P. patens ark* mutant^50^. Consistent with their ability of complementation, the Ser residue corresponding to the point mutation of the *ark* mutant is conserved in RAF4, RAF5, and RAF6 but not in other B3 Raf-like MAPKKKs^54^. In agreement with the ABA-insensitive seed germination in the series of triple knockout and knockdown mutants of *RAF3*, *RAF4*, and *RAF5*^51^, the triple mutant of *RAF4*, *RAF5*, and *RAF6* showed moderate ABA insensitivities in seedling establishment, stomatal closure, and gene expression yet greatly impaired responses to osmotic stress and desiccation^54^. These different sensitivities to ABA and osmotic stress appeared to be associated with the fact that the activation of subclass III SnRK2s by ABA was reduced in the triple mutant but that by osmotic stress was abolished^54^. In addition to RAF3, RAF4, and RAF5^51^, RAF6 phosphorylated SRK2E weakly compared to RAF4 and RAF5^54^ (Fig. 2). Noticeably, SRK2E was not able to phosphorylate RAF4^51,54^, whereas the SRK2E incubated with ABI1, which had reduced autophosphorylation activity, was activated by RAF4^54^. Collectively, B3 Raf-like MAPKKKs, RAF3, RAF4, RAF5, and RAF6, were shown to be the upstream kinases phosphorylating and activating ABA-responsive SnRK2s in response to ABA and osmotic stress.

Another study^53^ shed new light on the subclass I ABA-independent SnRK2s (Supplementary Table 1), which are little studied compared to the subclass III ABA-dependent SnRK2s. In vivo protein co-immunoprecipitation of subclass I SnRK2s followed by liquid chromatography-MS/MS identified candidate interacting proteins of SRK2A and SRK2G under osmotic stress conditions^53^. The common candidate interactors included three B4 Raf-like MAPKKKs (RAF18, RAF20, and RAF24), which have been shown to be phosphorylated in 5 min, in response to osmotic stress^99^. Importantly, previous phosphoproteomic analysis^99^ identified B4 Raf-like MAPKKKs, subclass I SnRK2, VCS, and DCP2 proteins to be rapidly phosphorylated by osmotic stress. In addition to the in vivo interaction of these three Raf-like MAPKKKs with SRK2A and SRK2G, further microscopic analyses revealed that RAF18, and SRK2A and SRK2G co-localized to P-bodies in response to osmotic stress, showing a punctuate pattern as previously reported for subclass I SnRK2s^76,77^. Furthermore, RAF18, RAF20, and RAF24 were shown to activate four subclass I SnRK2 members (SRK2A, SRK2B, SRK2G, and SRK2H) by phosphorylation in vitro (Fig. 2). Among several phosphorylation sites, Ser154 in SRK2G, the residue conserved in subclass I SnRK2s, was suggested to be directly phosphorylated by Raf-like MAPKKKs, consistent with the previous report that the residue in SRK2B was phosphorylated by osmotic stress^88^. In fact, the phosphorylation of SRK2G by itself or RAF20 was decreased by the substitution of Ser154 to Ala^53^. The in vivo kinase activity of subclass I SnRK2s in response to dehydration was significantly decreased in the *raf18 raf20 raf24* triple mutant plants, but not in the single or double mutant plants. By contrast, the activity of subclass III SnRK2s was not altered in the triple mutants, suggesting that the three RAF kinases have the redundant role in regulating specifically subclass I SnRK2s under osmotic stress conditions (Fig. 2). Consistent with previous reports showing that subclass I SnRK2s are involved in growth and mRNA decay under osmotic stress conditions^77^, the *raf18 raf20 raf24* triple mutants display growth retardation and altered gene expression similar to the *srk2a srk2b srk2g srk2h* quadruple mutants under stress conditions^53^, suggesting that the Raf-like MAPKKKs regulate the mRNA population under osmotic stress by activating subclass I SnRK2s. However, the *raf18 raf20 raf24* mutants showed slightly retarded growth compared with the *srk2a srk2b srk2g srk2h* mutants, implying that subclass I SnRK2s are main but not exclusive substrates of the three Raf-like MAPKKKs. In summary, B4 Raf-like MAPKKKs, RAF18, RAF20, and RAF24, were shown to regulate subclass I SnRK2-VCS signalling pathway under osmotic stress conditions but not ABA-responsive subclass III SnRK2s.

These new findings on B3 and B4 Raf-like MAPKKKs are supported by another study, in which two groups of protein kinases of ~130 and 100 kDa showed strong activation in response to short-term osmotic stress, but not ABA, even in the multiple mutants of SnRK2s^52^. The authors named these protein kinases OKs (osmotic stress-activated protein kinases). Further phosphoproteomic and mutational analyses revealed that the OK^130^ and OK^100^ groups correspond to B4 and B2/B3 Raf-like MAPKKKs, respectively (Supplementary Table 2). Consistent with the functional redundancy among RAF18, RAF20, and RAF24^53^, the single mutants of the B4 family of Raf-like MAPKKKs did not show significant changes on the kinase activity^52^. However, the high-order mutants generated by using CRISPR/Cas9-mediated genome editing for all members of B4 Raf-like MAPKKKs (Supplementary Table 2) in the *raf16* T-DNA background (OK^130^-weak and OK^130^-null) showed greatly reduced activities of the B4 Raf-like MAPKKKs and subclass I SnRK2s^52^. In particular, the activity of subclass I SnRK2s in response to osmotic stress was completely abolished in the B4 null mutant. The B4 null mutants displayed severe growth and developmental effects, including seed production, and were hypersensitive to salt and osmotic stresses, similar to the *raf18 raf20 raf24* triple mutants^53^. Altogether, these results support a redundant upstream regulation of subclass I SnRK2s by the B4 family of Raf-like MAPKKKs. By contrast, the activation of subclass III SnRK2s by ABA and osmotic stress was not altered in the B4 null mutant, suggesting that ABA-responsive SnRK2s are regulated independently of the B4 Raf-like MAPKKKs (Fig. 2). The high-order mutant for the B2/B3 families of Raf-like MAPKKKs, *raf2 raf4 raf5 raf10 raf11* quintuple mutant, showed reduced activity of subclass III SRK2D, SRK2E and SRK2I members under osmotic stress and strong plant growth inhibition phenotypes as well^52^. Furthermore, in the quindec mutant of B2/B3 Raf-like MAPKKKs generated in the B4 weak mutant background (*raf16 raf40 raf24 raf18 raf20 raf35 raf42 raf2 raf3 raf4 raf5 raf10 raf11*), the kinase activity of subclass III SnRK2 was greatly reduced or abolished by ABA and osmotic stress, respectively. Consistently, the quatdec mutant in the B4 weak mutant (*raf16 raf40 raf24 raf18 raf20 raf35 raf42 raf3 raf4 raf5 raf10 raf11*), which has no mutations in RAF2 and shows minor growth defects under control conditions unlike the quindec mutant, resulted in increased water loss and decreased ABA sensitivities in seed germination, seedling establishment, and gene expression compared to wild-type plants and other OK mutants lacking either B2/B3 or B4 Raf-like MAPKKKs. Although B4 Raf-like MAPKKKs RAF24 and RAF40 phosphorylated and activated subclass I SRK2A and SRK2B, several B2 and B3 family members, including RAF6, RAF7, and RAF10, phosphorylated subclass III SRK2E^52^ (Fig. 2). Previously, RAF10 from B2 Raf-like MAPKKKs was reported to be involved in ABA signalling by phosphorylating subclass III SnRK2s and their substrates, including ABI5^100^. Although the role of B3 Raf-like MAPKKKs was repeatedly reported^51,52,54^, further studies are needed to reveal how B2 and B3 Raf-like MAPKKKs function together in ABA and osmotic-stress signalling.

**Fig. 3:**
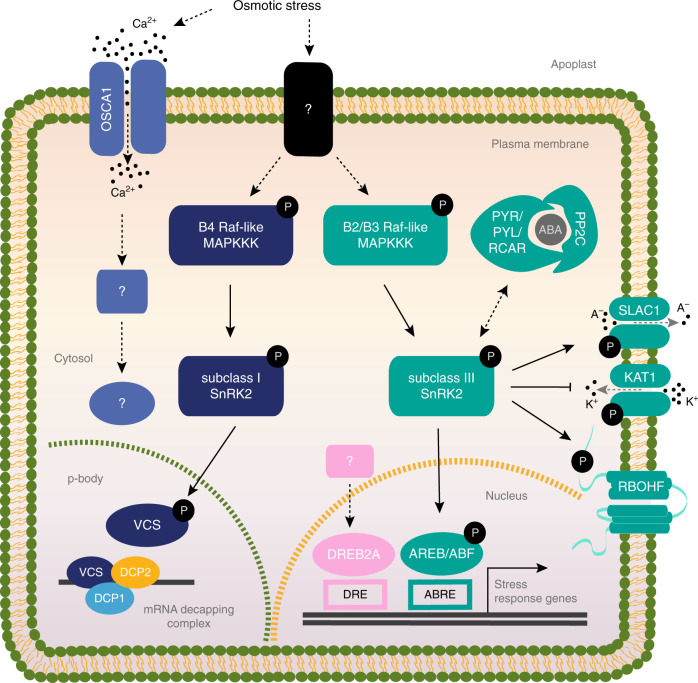
Current model of osmotic stress kinases network and signalling pathways. Osmotic stress evokes rapid increase of Ca^2+^, which is perceived by OSCA1 osmosensor proteins localized at plasma membrane. Question marks in violet objects depict possible downstream components activated by OSCA1 in response to osmotic stress. Raf-like kinases are activated in response to osmotic stress likely via other uncharacterized osmosensor proteins depicted as question mark in black box. B4 Raf-like MAPKKKs phosphorylate and activate subclass I SnRK2s, which are not responsive to ABA, and in turn SnRK2s phosphorylate mRNA binding proteins, such as VCS, which forms a protein complex with the decapping proteins DCP1 and DCP2 and are involved in post-transcriptional regulation of mRNA in p-bodies. B2/B3 Raf-like MAPKKKs phosphorylate and activate subclass III SnRK2s, which are ABA-responsive. In the presence of ABA, PYR/PYL/RCAR receptors form protein complexes with PP2C, resulting in the release of subclass III SnRK2s from inhibition by PP2Cs. Subclass III SnRK2s, phosphorylate SLAC1, KAT1 and RBOHF membrane proteins, which are essential for stomatal aperture regulation. Subclass III SnRK2s phosphorylate AREB/ABF transcription factors, which in turn activate the expression of stress-responsive genes. DREB2A transcription factor is activated through an ABA-independent signalling pathway. Question mark pink box depicts plausible upstream regulators of DREB2A still unknown. Straight arrows indicate direct interaction and phosphorylation, dashed arrows (black) indicate putative interactions not fully experimentally validated in vivo, and dashed arrows (grey) depict ion flux through membrane channel proteins. Double arrowhead dashed arrow indicates association–dissociation of PP2C with SnRK2s in the absence–presence of ABA.

In conclusion, these four studies^51–54^ elucidated novel and specific roles for B2/B3 family of Raf-like MAPKKKs in regulating the ABA signalling pathway by phosphorylation of the SnRK2s from subclass III and, for B4 Raf-like kinases, regulating the ABA-independent signalling pathway by phosphorylation of the SnRK2s from subclass I (Fig. 3).

### Self-activation of SnRK2s

Although SnRK2s were reported to be phosphorylated by RAF10 and BRASSINOSTEROID INSENSITIVE 2 kinases^100,101^, it has been accepted that once released from PP2Cs in response to ABA, subclass III SnRK2s are self-activated by autophosphorylation^38,39^. The studies reviewed herein suggest that Raf-like MAPKKKs mainly phosphorylate SnRK2s. Two serine residues in the activation loop, Ser171 and Ser175, are crucial for activation of SRK2E kinase^88^. MS analyses further revealed that RAF3 phosphorylates Ser171 of SRK2E^51^. Consistently, the substitutions Ser171Ala and/or Ser175Ala in SRK2E resulted in the loss of phosphorylation by ABA^51^ and by RAF4^54^, indicating that the conserved Ser residues in the activation loop of SRK2E are phosphorylated by RAF-like MAPKKKs. However, the ABA-responsive activation of SnRK2s was decreased but not abolished in the multiple mutants of RAF3, RAF4, RAF5, or RAF6^51,54^ or the quintuple mutants of RAF2, RAF4, RAF5, RAF10, and RAF11 B2/B3 Raf-like MAPKKKs^52^. Moreover, quintuple *raf2 raf4 raf5 raf10 raf11* mutants showed moderate insensitivities to ABA compared to the triple *srk2d srk2e srk2i* mutants^51,52,54^, implying that the ABA signalling pathway is still active to some extent, presumably because of the activation of SnRK2s by dissociation from PP2Cs, remaining members of MAPKKKs, and/or any other kinases. A hint to address the kinetic mechanisms of the inhibition and activation of SnRK2 by PP2Cs and Raf-like MAPKKKs, respectively, was provided by a recent report showing that group-A PP2Cs inhibit both subclass I and III SnRK2s^102^. Although group-A PP2C were thought to interact and dephosphorylate ABA-responsive SnRK2s, ABA-unresponsive SRK2A was shown to be inhibited by ABI1-type PP2Cs but not PP2CA-types, both of which inhibited SRK2E^102^, consistent with another report^103^. Further, fluorescence life-time imaging (Förster resonance energy transfer) in tobacco leaves revealed that RCAR receptors did not disrupt the interaction of ABI1 with SRK2E but SRK2A in the absence or low concentration of ABA, implying that the PP2Cs have different affinities to subclass I and III SnRK2s. Taken together, subclass III SnRK2s may require both dissociation from PP2Cs in response to ABA and activation by B2/B3 Raf-like kinases for full activation, whereas subclass I SnRK2 may be fully activated by B4 Raf-like kinases even before the accumulation of ABA.

### Crosstalk mechanisms of ABA-dependent and -independent pathways

Osmotic stress can be divided into the ABA-dependent and the ABA-independent responses. However, many experimental evidences indicate that these pathways are not lineal and independent but functionally interact with one another. The crosstalk between ABA-dependent and -independent pathways can occur at different levels. Even though the B2/B3 and B4 Raf-like MAPKKKs activate different subclasses of SnRK2s, the activation of Raf-like MAPKKKs is unlikely independent. In the B4 mutants, the activation of B2/B3 Raf-like MAPKKKs were partially impaired^52^, implying that B4 Raf-like MAPKKKs are involved in the regulation of the B2/B3 Raf-like MAPKKKs in response to osmotic stress. It still remains to be understood whether Raf-like MAPKKKs directly regulate each other or upstream proteins are involved in the control of the MAPKKKs in a connected way. B4 and B2/B3 Raf-like MAPKKKs showed preferences to phosphorylate subclass I and III SnRK2s, respectively. However, weak phosphorylation events of SRK2E by B4 Raf-like MAPKKKs and those of subclass I SnRK2s by B2/3 Raf-like MAPKKKs have been reported^51–54^ (Fig. 2a), supporting that these signalling pathways are indeed not lineal.

Evidences for crosstalk were also reported at the level of SnRK2s. As proven by the crystal structure^38^, subclass III SnRK2s interact with PP2Cs in a similar manner to the RCAR-PP2C ABA coreceptors. By contrast, ABI1-type PP2Cs were reported to be functional phosphatases of SnRK2 regardless of subclasses^102,103^. Moreover, the substrates of SnRK2s might be regulated in response to both ABA and osmotic stress. Although the large overlap of the downstream genes indicated that subclass III SnRK2s are the main activators of AREB/ABFs^33^, AREB/ABFs were also activated by subclass I and II SnRK2s^74,102^. Furthermore, the phosphorylation of VCS appeared to be downregulated in the *srk2d srk2e srk2i* mutants^66^ as well as in *srk2a srk2b srk2g srk2h* mutants^77^. Unlike subclass I SnRK2s, SRK2D did not show interaction with VCS in planta, whereas the slight in vivo phosphorylation of VCS by SRK2D in response to dehydration was reported^77^. The transcription factors downstream of the SnRK2s, including AREB/ABFs and DREB2A, also function interdependently^21^. In summary, many evidences indicate that ABA-dependent and -independent pathways might converge at the SnRK2s, as well as in their upstream and downstream factors.

### Evolution of osmotic stress signalling pathways

Plants have acquired molecular and physiological mechanisms to adapt osmotic and drought tolerance traits, which were critical in the evolution of plants from water to land. Recently reported genomes of charophyte algae, members of Zygnematophyceae as the sister linage to land plant, provided insights into the terrestrial adaptation^104,105^. In particular, RCAR/PYR/PYL ABA receptors were suggested to be acquired by horizontal gene transfer from soil bacteria^104^, likely consistent with the presence of a gene corresponding to RCAR/PYR/PYL in streptophyte algae^106^. Moreover, the ancestral type of the receptor in *Zygnema circumcarinatum* alga inhibited the phosphatase activity of PP2C independently of ligand, i.e. ABA, suggesting that the property of ABA binding was acquired during evolution from algae to land plants^107^. By contrast, *Klebsormidium nitens* (*K. nitens*) a semi-terrestrial green alga, have all the components of ABA signalling, except for the ABA receptors^108^. Nonetheless, the *K. nitens* SnRK2 kinase, which is closer to subclass III SnRK2s in *Arabidopsis*, restored the downstream pathways in response to ABA in *Arabidopsis*^109^ and *P. patens*^85^. In addition to the *K. nitens* SnRK2 kinase, *Arabidopsis* SRK2C (subclass II) and SRK2E (subclass III) kinases restored the ABA- and osmotic stress-responses in the null mutant of *PpSnRK2*s, whereas SRK2H (subclass I) kinase did not^85^. Altogether, these studies suggest that subclass III is an ancient form of SnRK2s and the most recent form is subclass I emerged before angiosperms.

Vegetative desiccation tolerance is a typical trait of bryophytes to survive in extreme water-deficit conditions that is not present in vascular plants. Both ABA treatment^110^ and downregulation of clade A PP2Cs^89^ promote *P. patens* survival in extreme desiccation conditions. Clade A PP2C act downstream of ABA-activated SnRK2s in *P. patens* and it has been hypothesized that they emerged to repress ABA-mediated desiccation tolerance, facilitating the vegetative propagation of land plants^89^. Although two genes encode for clade A PP2C in *P. patens*, which mainly regulate ABA-induced genes^89^, nine genes in the seed plant *Arabidopsis* genome may play different roles in different tissues and organs. Later in evolution, PYR/PYL/RCAR ABA receptors might be able to recruit PP2Cs, thus activating SnRK2s and improving growth and stress survival on land plants^89^.

Although ARK is the single gene representing the B3 Raf-like MAPKKKs in *P. patens*, this clade might be later expanded in angiosperms, as six B3-MAPKKK genes are encoded in *Arabidopsis* (Supplementary Table 2 and Fig. 2a). In particular, RAF4, RAF5, and RAF6, in which the Ser residue of the *ark* point mutation is conserved, appeared to be the functional orthologs of ARK^50,54^. The current hypothesis is that ABA-B3 Raf-like MAPKKK-subclass III SnRK2 signalling module existing in *P. patens* has been conserved to protect plants from drought during evolution. Later, bryophytes plants evolved by acquiring a novel subclass I SnRK2 system in vascular plants that conferred further protection to osmotic stress independently from the ancient system^54,85^. Similarly, B4 Raf-like kinases might have been specifically acquired by vascular plants as well, suggesting that the signalling module B4 Raf-like MAPKKK-subclass I SnRK2s-VCS emerged to control post-transcriptional functions and improve the stress-adaptive response of plants against osmotic stress^77^. As some B4 Raf-like kinases family members are not conserved in *P. patens*, specific members of the B4 Raf-like kinases and subclass I SnRK2s might have been specifically acquired by seed plants to improve the adaptation mechanisms of these plants in osmotic stress conditions. Consistently, the RAF18/20/24-subclass I SnRK2-VCS signalling cascade is activated in response to osmotic stress and not ABA^53^. In summary, land plants have developed new molecular mechanisms for perception and signal transduction in response to osmotic stress, which have been crucial to confer protection to higher plants against drought.

### Outlook and future perspectives

Although the ABA activation of SnRK2s and the ABA-core signalling pathway were well studied, Raf-like MAPKKKs have been just recently identified to be crucial for the osmotic stress-mediated activation of SnRK2s^50–54^. Importantly, these studies report the role of Raf-like MAPKKKs in early osmotic stress signalling controlling both ABA-dependent and ABA-independent pathways in higher plants^51–54^. Thus, Raf-like kinases are likely connecting the osmosensing machinery of plants to SnRK2s (Fig. 3). However, the osmosensor components upstream Raf-like MAPKKKs still need to be identified. The structural comparison with well-studied Raf-like MAPKKK, such as CTR1 interacting with ethylene receptors^111^, may provide insights into the upstream sensory proteins and the substrate specificities for SnRK2s. Importantly, Raf-like MAPKKKs seem to function independently from the OSCA1 osmosensor^52^. Besides the Raf-like MAPKKKs, an increasing number of proteins have been reported to regulate SnRK2s post-translationally^112–117^. Future studies addressing how Raf-like MAPKKs function together with OSCA1 and other upstream factors of SnRK2s in response to osmotic stress should shed new light on this direction.

Here we connect the Raf-like MAPKKKs with the state-of-the-art knowledge offering general schematic picture of the osmotic regulation of ABA-dependent and -independent pathways (Fig. 3). Still, many remaining questions need to be addressed: (i) How plants sense osmotic stress? (ii) What is the signal that leads to the triggering of the osmotic stress signalling pathways? (iii) What are the molecular and physiological functions of ABA-independent pathways in response to osmotic stress? Further studies in this direction are needed to continue deciphering the osmotic stress signalling pathways and improving our understanding on how plants cope with water-deficit stress.

## Supplementary information

Supplementary Information
