# Supplementary material for: Role of Raf-like kinases in SnRK2 activation and osmotic stress response in plants
Source: Nat Commun. 2020 Dec 3;11:6184. doi: 10.1038/s41467-020-19977-2 (PMC7712759; doi:10.1038/s41467-020-19977-2)
Supplement: Supplementary file 1 — Supplementary Information [file 41467_2020_19977_MOESM1_ESM.pdf]

Supplementary Table 1. Classification of Arabidopsis SnRK2 kinases

AGI code	Subclass	Gene name (alphabetical)	Gene name (numerical)	Alias	Activation by ABA[1]	Activation by osmotic stress[1]	Subcellular localization[2-5]	Re-localization by osmotic stress or ABA[2,3,5]
AT1G10940	I	SRK2A	SnRK2.4		no	yes	Cytoplasm	P-bodies
AT1G60940	I	SRK2B	SnRK2.10		no	yes	Cytoplasm	n.d.
AT5G08590	I	SRK2G	SnRK2.1		no	yes	Cytoplasm, nuclei	P-bodies
AT5G63650	I	SRK2H	SnRK2.5		no	yes	n.d.	n.d.
AT2G23030	I	SRK2J	SnRK2.9		no	no	n.d.	n.d.
AT1G78290	II	SRK2C	SnRK2.8		yes	yes	Cytoplasm, nuclei	n.d.
AT4G40010	II	SRK2F	SnRK2.7		yes	yes	Cytoplasm, nuclei	n.d.
AT3G50500	III	SRK2D	SnRK2.2		yes	yes	Cytoplasm, nuclei	Nuclei
AT4G33950	III	SRK2E	SnRK2.6	OST1	yes	yes	Cytoplasm, nuclei	n.d.
AT5G66880	III	SRK2I	SnRK2.3		yes	yes	Cytoplasm, nuclei	n.d.

n.d., no data available.

Supplementary Table 2. Classification of Arabidopsis Raf-like MAPKKK B subfamily members

AGI code	Subfamily	OK subfamily [6]	Gene name [7,*]	Gene name [8]	Gene name [9]	Alias
AT2G31010	B1		RAF13	M3Kø1		
AT2G42640	B1		RAF14	M3Kø2		
AT3G58640	B1		RAF15			
AT3G06620	B2	OK^{100}	RAF7			
AT3G06630	B2	OK^{100}	RAF8			
AT3G06640	B2	OK^{100}	RAF9			
AT5G49470	B2	OK^{100}	RAF10	M3Kη3		
AT1G67890	B2	OK^{100}	RAF11			
AT4G23050	B2	OK^{100}	RAF12	M3K∂4		
AT5G03730	B3	OK^{100}	RAF1			CTR1, SIS1
AT1G08720	B3	OK^{100}	RAF2	M3K∂3		EDR1
AT5G11850	B3	OK^{100}	RAF3	M3K∂1		
AT1G18160	B3	OK^{100}	RAF4	M3K∂7	AtARK1	
AT1G73660	B3	OK^{100}	RAF5	M3K∂6	AtARK2	SIS8
AT4G24480	B3	OK^{100}	RAF6	M3K∂5	AtARK3	
AT1G04700	B4	OK^{130}	RAF16			
AT1G16270	B4	OK^{130}	RAF18			
AT1G79570	B4	OK^{130}	RAF20			
AT2G35050	B4	OK^{130}	RAF24			
AT5G57610	B4	OK^{130}	RAF35			
AT3G24715	B4	OK^{130}	RAF40			HCR1
AT3G46920	B4	OK^{130}	RAF42			

OK, osmotic stress-activated protein kinases

*Arabidopsis MAPKKK Gene Family in the TAIR database: https://www.arabidopsis.org/browse/genefamily/MAPKKK.jsp

Supplementary References

1. Boudsocq, M., Barbier-Brygoo, H. & Lauriere, C. Identification of nine sucrose nonfermenting 1-related protein kinases 2 activated by hyperosmotic and saline stresses in Arabidopsis thaliana. *J Biol Chem* **279**, 41758–41766 (2004).

2. McLoughlin, F. *et al.* The Snf1-related protein kinases SnRK2.4 and SnRK2.10 are involved in maintenance of root system architecture during salt stress. *Plant J* **72**, 436–449 (2012).

3. Soma, F. *et al.* ABA-unresponsive SnRK2 protein kinases regulate mRNA decay under osmotic stress in plants. *Nat Plants* **3**, 16204 (2017).

4. Mizoguchi, M. *et al.* Two closely related subclass II SnRK2 protein kinases cooperatively regulate drought-inducible gene expression. *Plant Cell Physiol* **51**, 842–847 (2010).

5. Fujita, Y. *et al.* Three SnRK2 protein kinases are the main positive regulators of abscisic acid signaling in response to water stress in Arabidopsis. *Plant Cell Physiol* **50**, 2123–2132 (2009).

6. Lin, Z. *et al.* A RAF-SnRK2 kinase cascade mediates early osmotic stress signaling in higher plants. *Nat Commun* **11**, 613 (2020).

7. Mapk-Group. Mitogen-activated protein kinase cascades in plants: a new nomenclature. *Trends Plant Sci* **7**, 301–308 (2002).

8. Takahashi, Y. *et al.* MAP3Kinase-dependent SnRK2-kinase activation is required for abscisic acid signal transduction and rapid osmotic stress response. *Nat Commun* **11**, 12 (2020).

9. Katsuta, S. *et al.* Arabidopsis Raf-like kinases act as positive regulators of subclass III SnRK2 in osmostress signaling. *Plant J* (2020). doi:10.1111/tpj.14756
